# Discovery of New Inhibitors of Toxoplasma gondii via the Pathogen Box

**DOI:** 10.1128/AAC.01640-17

**Published:** 2018-01-25

**Authors:** Jérémy Spalenka, Sandie Escotte-Binet, Ali Bakiri, Jane Hubert, Jean-Hugues Renault, Frédéric Velard, Simon Duchateau, Dominique Aubert, Antoine Huguenin, Isabelle Villena

**Affiliations:** a Laboratoire de Parasitologie–Mycologie, EA 3800, SFR CAP-Santé FED 4231, Centre Hospitalier de Reims et Université de Reims Champagne-Ardenne, Reims Cedex, France; b UMR CNRS 7312, Université de Reims Champagne-Ardenne, Reims Cedex 2, France; c Biomatériaux et Inflammation en site Osseux, EA 4691, Université de Reims Champagne-Ardenne, Reims Cedex, France; d Centre National de Référence de la Toxoplasmose, Centre de Ressources Biologiques Toxoplasma, EA 3800, SFR CAP-Santé FED 4231, Centre Hospitalier de Reims et Université de Reims Champagne-Ardenne, Reims Cedex, France

**Keywords:** Toxoplasma gondii, drug screening, antitoxoplasmic compound, Pathogen Box, antitoxoplasmic activity

## Abstract

Toxoplasma gondii is a cosmopolitan protozoan parasite which affects approximately 30% of the population worldwide. The drugs currently used against toxoplasmosis are few in number and show several limitations, such as drug intolerance, poor bioavailability, or drug resistance mechanism developed by the parasite. Thus, it is important to find new compounds able to inhibit parasite invasion or proliferation. In this study, the 400 compounds of the open-access Pathogen Box, provided by the Medicines for Malaria Venture (MMV) foundation, were screened for their anti-Toxoplasma gondii activity. A preliminary *in vitro* screening performed over 72 h by an enzyme-linked immunosorbent assay (ELISA) revealed 15 interesting compounds that were effective against T. gondii at 1 μM. Their cytotoxicity was estimated on Vero cells, and their 50% inhibitory concentrations (IC_50_) were further calculated. As a result, eight anti-Toxoplasma gondii compounds with an IC_50_ of less than 2 μM and a selectivity index (SI) value of greater than 4 were identified. The most active was MMV675968, showing an IC_50_ of 0.02 μM and a selectivity index value equal to 275. Two other compounds, MMV689480 and MMV687807, also showed a good activity against T. gondii, with IC_50_s of 0.10 μM (SI of 86.6) and 0.15 μM (SI of 11.3), respectively. Structure-activity relationships for the eight selected compounds also were discussed on the basis of fingerprinting similarity measurements using the Tanimoto method. The anti-Toxoplasma gondii compounds highlighted here represent potential candidates for the development of new drugs that could be used against toxoplasmosis.

## INTRODUCTION

Toxoplasmosis is one of the most important parasitic diseases worldwide. It is caused by the protozoan Toxoplasma gondii and affects approximately 25 to 30% of the world population (1). Toxoplasma gondii is able to cause severe illness that can be life-threatening in immunocompromised individuals or in fetuses when acquired congenitally (2). Moreover, ocular lesions can occur in the case of congenital toxoplasmosis or during reactivation of toxoplasmosis. For example, the proportion of people with ocular toxoplasmosis in the United States is about 2%, and this number can be much greater in other countries, especially in South America (3). Only a few treatments against toxoplasmosis are currently available, mainly consisting of a synergic combination of pyrimethamine and sulfonamide. These two drugs block the folate biosynthesis pathway by inhibiting two enzymes which are essential for parasite survival and growth, namely, dihydropteroate synthase (DHPS) and dihydrofolate reductase (DHFR). However, treatment of toxoplasmic encephalitis and chorioretinitis by these drugs can fail due to intolerance, poor absorption of these molecules (4, 5), or parasite resistance (6, 7). For these reasons, it is very important to search for new active compounds against toxoplasmosis.

The Medicines for Malaria Venture (MMV) foundation, which aims to reduce the burden of malaria by developing and facilitating the delivery of new drug candidates (http://www.mmv.org/research-development), recently was made available free of charge for all scientists as the so-called Pathogen Box. It was modeled on the open-access Malaria Box that has been widely used by several teams on different types of pathogens, such as cancer cells, protozoans, and bacteria (8–10). Each of the 400 compounds in the Pathogen Box has confirmed activity against pathogens that cause some of the most socioeconomically important diseases worldwide, including tuberculosis, malaria, sleeping sickness, leishmaniasis, schistosomiasis, hookworm disease, toxoplasmosis, cryptosporidiosis, and dengue. The compounds within Pathogen Box have been tested for cytotoxicity, with compounds included in the library being at least 5-fold more selective for the pathogen than its mammalian host. Included in the 400 compounds of the Pathogen Box is a set of 26 reference compounds with activity against at least one of these pathogens. MMV has also provided the biological activity of compounds from screening platforms (ChEMBL-NTD; https://www.ebi.ac.uk/chemblntd), the plate layout, and compounds details (structures, trivial names, salt forms, and cLogP). These data can be found in an Excel spreadsheet, referred to as the Pathogen Box supporting information (https://www.pathogenbox.org/about-pathogen-box/supporting-information).

The Pathogen Box is a powerful tool that could lead to the synthesis of new active molecules based on the structures of its compounds and improve the therapeutic armamentarium.

In this study, all compounds provided in the MMV foundation's Pathogen Box were screened by following the work that was done before with the Malaria Box on T. gondii (8, 11) and Cryptosporidium parvum (12).

## RESULTS

### Screening on T. gondii.

In this study, all compounds provided in the MMV foundation's Pathogen Box were screened on T. gondii tachyzoites. At first the 400 compounds of the Pathogen Box were screened at a single concentration of 1 μM in order to select the potential antiparasitic candidates before performing further IC_50_ and 50% cytotoxic concentration (CC_50_) measurements. The 15 compounds reported in Table 1 demonstrated significant antiparasitic activity, as revealed by their ability to inhibit at least 50% of T. gondii growth at 1 μM. These results were confirmed by microscopic observations.

**TABLE 1:** Characteristics of the 15 compounds showing antitoxoplasmic activity after preliminary screening at 1 μM

Anti-Toxoplasma gondii compound^*a*^	Mol wt^*b*^	cLogP^*b*^	Target^*b*^
MMV676477	383.47	3.28	Tuberculosis
MMV676512	347.39	2.95	Tuberculosis
MMV676604	371.46	2.11	Kinetoplastids
MMV688853	389.88	1.75	Cryptosporidiosis
MMV689480	326.43	4.69	Leishmaniasis
MMV676602	460.57	2.09	Kinetoplastids
MMV687807	383.67	2.63	Tuberculosis
MMV011765	358.73	3.10	Malaria
MMV022478	545.93	2.55	Malaria
MMV675968	359.81	2.31	Cryptosporidiosis
MMV659004	364.88	4.39	Kinetoplastids
MMV658988	338.84	3.93	Kinetoplastids
MMV676599	331.41	3.36	Cryptosporidiosis
MMV021013	294.40	3.55	Tuberculosis
MMV688703	335.42	4.03	Toxoplasmosis

a Compounds are named by their MMV identifier codes.

b Molecular weight (Mol wt), cLogP values, and initial activities of the compounds were obtained from the Pathogen Box supporting information.

### Cytotoxicity on Vero cells.

The cytotoxicity of the 15 compounds that were efficient against T. gondii according to the preliminary screening assay was assessed. We found that CC_50_ values ranged from 1.69 μM to 15.92 μM, depending on the compound we tested (Table 2).

**TABLE 2:** Characteristics of the eight compounds showing antitoxoplasmic activity according to our hit criteria with pyrimethamine as reference drug^*f*^

Anti-Toxoplasma gondii compound^*a*^	Mol wt^*b*^	cLogP^*b*^	IC_50_^*c*^ (μM)	CC_50_^*d*^ (μM)	SI^*e*^
MMV675968	359.81	2.31	0.02 ± 0.002*	5.5	275
MMV689480	326.43	4.69	0.10 ± 0.049*	8.66	86.6
MMV687807	383.67	2.63	0.15 ± 0.021*	1.69	11.3
MMV022478	545.93	2.55	0.29 ± 0.021*	2.23	7.7
MMV011765	358.73	3.10	0.34 ± 0.007*	9.48	27.9
MMV676602	460.57	2.09	0.81 ± 0.099*	3.30	4.1
MMV676512	347.39	2.95	0.86 ± 0.113*	3.61	4.2
MMV021013	294.40	3.55	1.12 ± 0.035	15.92	14.2
PYR	248.71	3.00	1.17 ± 0.076	10.52	9.0

a Compounds are named by their MMV identifier codes. PYR, pyrimethamine.

b Molecular weight and cLogP values were obtained from Pathogen Box supporting information, except for pyrimethamine, for which data were found on http://DrugCentral.org.

c Compounds were diluted by a 2-fold dilution series and tested in cell culture. Results are means from four values from two different experiments. *, *P* < 0.05 compared to values for PYR.

d Cytotoxicity against Vero cells was evaluated in cell culture. Results are means from four values from two different experiments.

e Selectivity indexes were calculated based on the CC_50 Vero cells_/IC_50_ *_T. gondii_* ratio.

f Hit criteria were an IC_50_ of <2 μM and SI of >4.

### Chemosensibility of T. gondii.

**FIG 1:**
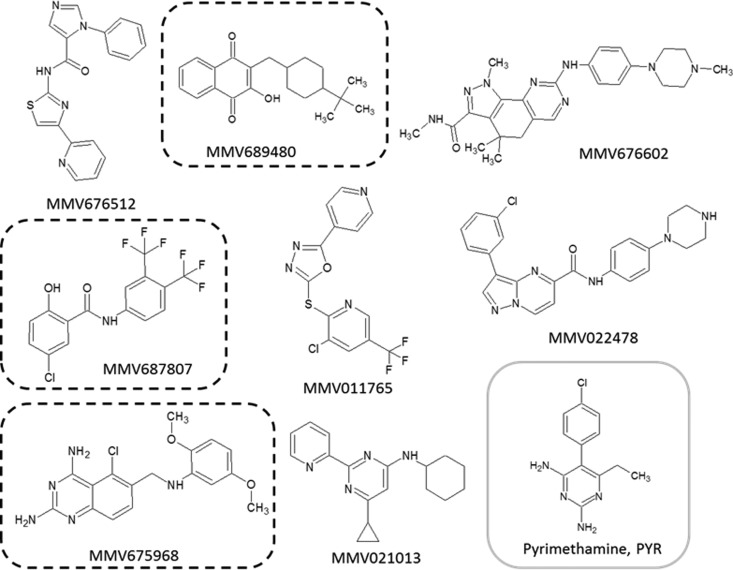
Structures of the compounds showing a selective antitoxoplasmic activity. The structures encircled by dotted lines highlight the most active compounds. These structures and the MMV identifiers were provided by the MMV foundation as supporting information for the open-access Pathogen Box.

**FIG 2:**
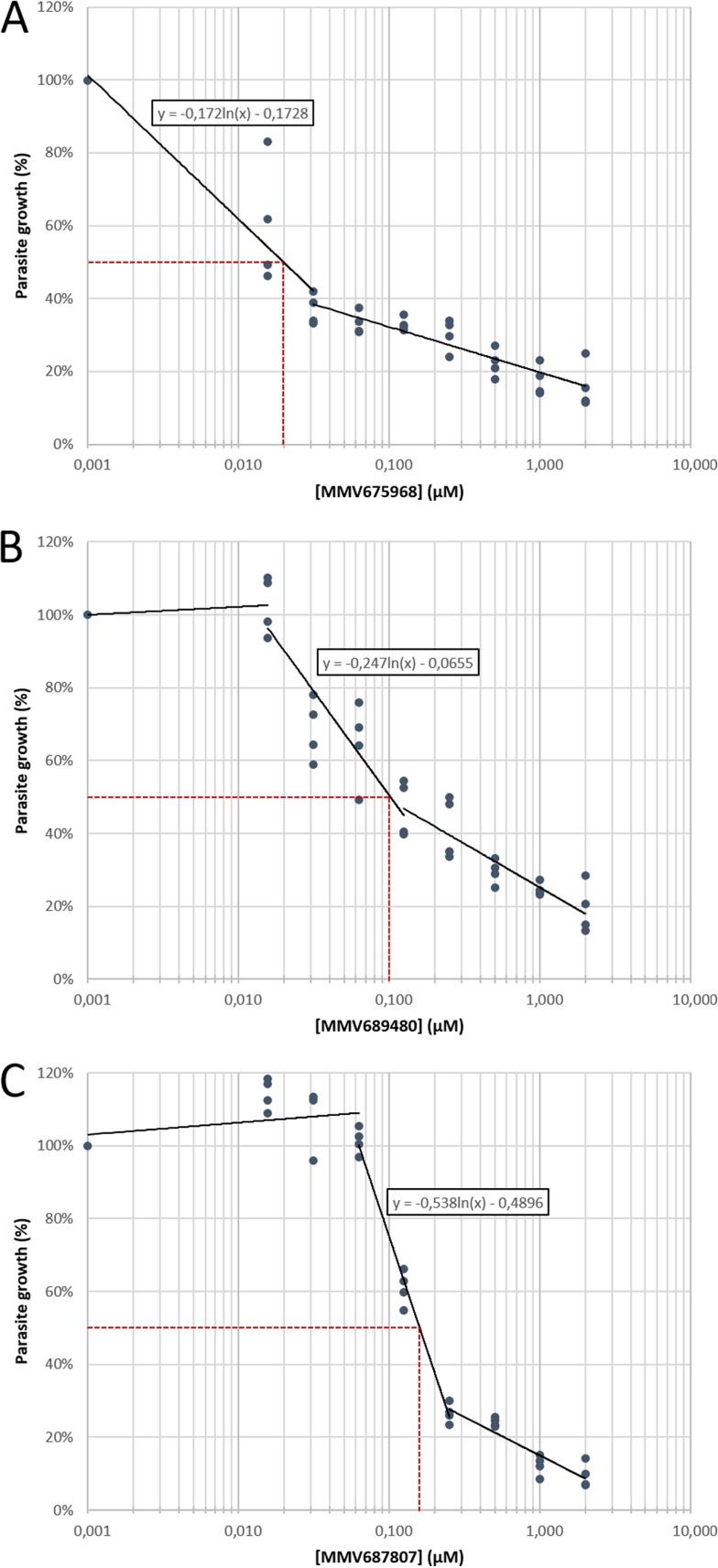
Representative figures of dose-response curves of the three most active compounds, MMV675968 (A), MMV689480 (B), and MMV687807 (C), against T. gondii. Concentrations ranged from 0 to 2 μM. Results were obtained from two different experiments consisting of two replicates per condition. Each dot represents one replicate value. The dotted line indicates 50% reduction in parasite growth.

Among the 15 compounds of the Pathogen Box that inhibited the growth of T. gondii at 1 μM by our screening method and based on our hit criteria (IC_50_, <2 μM; SI, >4), eight compounds showed a selective antitoxoplasmic activity: MMV676512, MMV689480, MMV676602, MMV687807, MMV011765, MMV022478, MMV675968, and MMV021013 (Table 2). The chemical structures of these eight selective compounds are shown in Fig. 1. Among them, MMV675968 (Fig. 2A), MMV689480 (Fig. 2B), and MMV687807 (Fig. 2C) were very active (IC_50_ of 0.02 μM, 0.10 μM, and 0.15 μM, respectively) (Table 2), thus providing interesting potential as anti-T. gondii drug candidates. On the contrary, the other seven compounds were not selective, including MMV688703.

### Structural similarity analysis.

**FIG 3:**
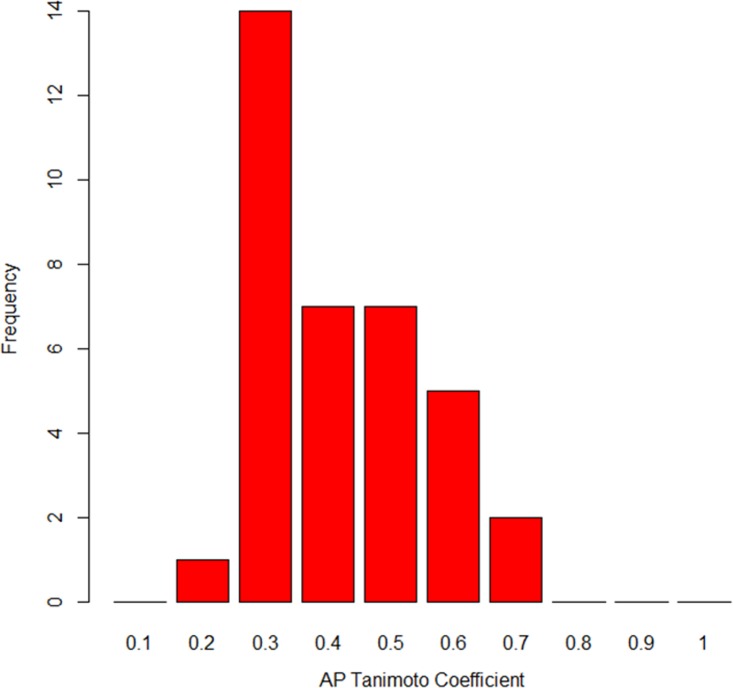
Frequency of the Tanimoto coefficient values between the nine molecules, including pyrimethamine, based on their atom pair fingerprints.

According to the similar property principle, compounds with similar chemical structures tend to have similar biological properties (13). In order to evaluate the structural similarities between the eight most active molecules together with the positive reference pyrimethamine (PYR), a Tanimoto coefficient (Tc) was calculated for every pair of the nine molecules on the basis of their atom pair (AP) fingerprint. Tc takes values of 0 to 1, from the least similar to the most similar. Figure 3 represents the bar plots of the frequency of Tc values between the nine molecules.

**FIG 4:**
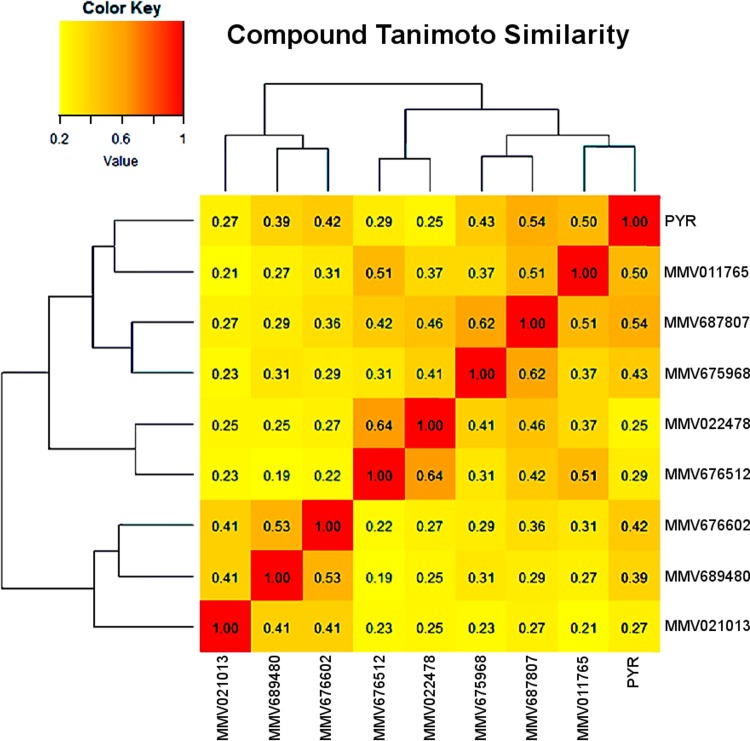
Hierarchical clustering analysis highlighting the structural similarities between the nine molecules, including pyrimethamine (PYR), according to their Tanimoto coefficients.

The most frequent values were between 0.3 and 0.5. These values are relatively low, indicating a relatively high structural diversity within the set of the nine molecules. The highest value was 0.64 between the compounds MMV022478 and MMV676512. In a second step, the resulting Tc similarity matrix was submitted as a distance matrix to hierarchical clustering analysis (HCA). HCA was carried out to highlight structural similarities among the eight active compounds together with pyrimethamine (PYR). Figure 4 reveals three main clusters. The first cluster contains the positive-control PYR along with MMV011765, MMV675968, and MMV687807, which has the highest Tc to PYR (0.54). Compounds MMV675968 and MMV687807, which are among the 3 most active molecules, also were included in this first cluster, whereas the third compound (MMV689480), with a high antitoxoplasmic activity, was located in the third cluster and exhibited a very low Tc compared to the two others (0.31 and 0.29, respectively). These results give insight into the structural similarities between the subset of active compounds; however, deeper analysis would be necessary for a more detailed structure-activity relationship evaluation.

## DISCUSSION

Toxoplasmosis ranks as one of the world's most common and neglected diseases induced by a protozoan parasite (14). The treatments currently used are not numerous, and most of them were discovered several decades ago (15, 16). Chemotherapy for treating toxoplasmosis frequently consists of combination treatments, usually the association of the antifolates pyrimethamine and sulfadiazine. To make things worse, the parasite can show a variable susceptibility toward the drugs (7) or even develop a resistance against one of those drugs (6). An ideal anti-Toxoplasma drug would be potent and nontoxic and would eliminate latent infection (bradyzoites).

The same issues can be found with Plasmodium falciparum (17). This is why, at first, the Medicines for Malaria Venture foundation and several groups of scientists decided to collaborate in order to propose a powerful screening tool, named Malaria Box (9). This tool was used to screen drugs on other pathogens, such as T. gondii, Entamoeba histolytica (8), Cryptosporidium parvum (12), Schistosoma (18), and Perkinsus marinus (19), among others. Fifty-five groups compiled more than 290 assay results describing the many activities of the Malaria Box compounds (9). For Toxoplasma, seven anti-Toxoplasma compounds with a 50% inhibitory concentration (IC_50_) lower than 5 μM and selectivity indexes (SI) higher than 6 were identified (8). The results have ignited over 30 drug development programs for a variety of diseases. This open-access effort was so successful that the MMV foundation distributed another set of compounds: the Pathogen Box, a new screening tool dedicated to diverse pathogens (20, 21) and based on the same principle as the Malaria Box.

In our study, eight antitoxoplasmic compounds were identified (Fig. 1 and Table 2) based on our hit criteria (IC_50_ of <2 μM and SI of >4), meaning that 2% of the compounds of the Pathogen Box were effective against the parasite.

New successes from Pathogen Box have been published recently with the demonstration of inhibitory activity against planktonic growth and Candida albicans biofilm (22). MMV675968 has been shown to be efficient against the planktonic form of C. albicans; moreover, this compound is also the most active against Toxoplasma, with an IC_50_ of 0.02 μM and a selectivity index value equal to 275. Antifungal agents target a broad range of eukaryotic fungal pathogens of human, and azoles have shown effect against Toxoplasma. Although fluconazole and itraconazole have IC_50_s of 3 μM and 0.5 μM, respectively, the mechanism responsible for their effect against Toxoplasma gondii is unknown. Also described as an anti-Cryptosporidium, MMV675968 showed an antiplasmodial activity against P. falciparum (23). MMV675968 is known to target Cryptosporidium DHFR (24), and it is likely that this compound also targets Plasmodium and Toxoplasma gondii dihydrofolate reductase.

Interestingly, one of our selected compounds was a well-known reference compound: buparvaquone (MMV689480; IC_50_ of 0.10 μM and SI of 86.6). Buparvaquone was previously shown to inhibit Neospora caninum proliferation *in vitro* and *in vivo* (25) and Toxoplasma gondii proliferation *in vivo* (26). This is in accordance with our results, since buparvaquone showed one of the highest antitoxoplasmic activities in the present study. Neospora caninum belongs to the Apicomplexa phylum, like Toxoplasma gondii or Eimeria tenella. Interestingly, the effect of buparvaquone has been highlighted in this parasite: it inhibits several enzymes involved in mitochondrial electron transport (27). Thus, this mechanism also could be applied for the inhibition of T. gondii growth.

MMV687807 is active against preformed biofilms of Candida albicans (22). This antimycobacterium compound has shown good activity (IC_50_ of 0.15 μM and SI of 11.3) against Toxoplasma gondii. The anti-Plasmodium compounds MMV022478 (IC_50_ of 0.29 μM and SI of 7.7) and MMV011765 (IC_50_ of 0.34 μM and SI of 27.9) also were efficient against T. gondii. MMV022478 was identified as selective for Trypanosoma brucei brucei (23). Members of the pyrazolo[1,5-a]pyrimidine class, to which MMV022478 belongs, have been reported to inhibit mammalian NADPH oxidase 4 (28). MMV676602 anti-Trypanosoma and MMV676512 anti-Cryptosporidium also were active against T. gondii (IC_50_ of 0.81 μM and SI of 4.1 and IC_50_ of 0.86 μM and SI of 4.2).

The anti-Mycobacterium compound MMV021013 also was efficient against Toxoplasma gondii (IC_50_ of 1.12 and SI of 14.2). This 2-pyridil-4-aminopyrimidine was active against T. cruzi, L. donovani, and T. b. brucei (23). Duffy et al. proposed, based on chemical structure, that the cellular target of this compound is methionine aminopeptidase (23). This compound was the only one that did not show significant activity compared to that of pyrimethamine.

None of the compounds, except MMV688703, presented as anti-Toxoplasma in the Pathogen Box showed activity with our method. This could be due to the different techniques used and also by the time of action of the drugs (72 h in our case). Moreover, P. falciparum is phylogenetically distant from T. gondii, even if they both belong to the Apicomplexa phylum. This would explain why some anti-Plasmodium compounds are not active against T. gondii.

MV688703, which presented antiplasmodial activity (23), was included in the 15 compounds isolated by screening. Unfortunately, it did not show the criteria for inclusion as an active compound (IC_50_ <2 μM and SI >4). This product was previously identified as an active compound against Toxoplasma gondii by inhibition of Toxoplasma cGMP-dependent protein kinase, involved in the regulation of calcium (29). Van Voorhis et al. saw some discrepancies in the values obtained for the same compounds in similar assays that were carried out by multiple groups, such as activity against P. falciparum, Trypanosoma spp., and mammalian cells (9). Some of these apparent discrepancies probably were due to variations in the techniques used for the screenings (9) or the experimental models (30).

Finally, an important point to evaluate interest in new compounds is the comparison of their efficiency to that of pyrimethamine. Pyrimethamine is the reference drug commonly used, in combination with sulfamide, to obtain a synergistic effect against T. gondii (31). Nevertheless, a variability in the susceptibilities of T. gondii strains to pyrimethamine has been observed naturally (7), and resistance toward this drug has been induced *in vitro* (32). Moreover, natural resistant strains of Plasmodium falciparum have been highlighted in several countries (33). This problem could be avoided by the newly discovered active drugs, since their scaffolds are completely different from the scaffold of pyrimethamine, as shown in Fig. 4, which derives from a pyrimidine skeleton. It could lead to the synthesis of new active molecules based on these structures and improve the therapeutic armamentarium.

The MMV foundation's Pathogen Box is a very powerful tool that grants easy and fast identification of new antiparasitic compounds with a very interesting yield (2%).

## MATERIALS AND METHODS

### Pathogen Box compounds.

All tested compounds were obtained from the Medicines for Malaria Venture (MMV) foundation (Geneva, Switzerland). The Pathogen Box was supplied in 96-well plates. Each compound (one per well) was diluted in 10 μl of dimethyl sulfoxide (DMSO) at a concentration of 10 mM and shipped frozen. The compounds were diluted in the culture medium at 1 or 2 μM top concentration in accordance with MMV instructions.

### Toxoplasma gondii strain.

Tachyzoites of the RH strain (type I) used in this study were provided by the French Biological Toxoplasma Resource Centre (BRC Toxoplasma, Reims, France).

### Parasite growth.

Tachyzoites were cultured on Vero cell monolayers (ATCC CCL-81) at 37°C under 5% CO_2_ in a humidified incubator. Both cells and parasites were grown in the complete medium Iscove's modified Dulbecco's medium-GlutaMAX (IMDM) (Invitrogen, Paris, France) supplemented with 2% (vol/vol) fetal calf serum (Biowest, Nuaillé, France) and antibiotics (100 IU/ml penicillin and 0.1 mg/ml streptomycin) (GIBCO). Host cells were infected at a 1:1 parasite-to-cell ratio. Cells and tachyzoites were counted using a Kova Slide counting chamber with trypan blue. The parasites were routinely checked for Mycoplasma species contamination and found to be negative using a Mycoplasma species real-time PCR (34).

### Screening of the Pathogen Box compounds.

The 400 compounds of the Pathogen Box were prepared according to the MMV foundation instructions provided with the Box and screened on T. gondii in 96-well plates, using pyrimethamine (PYR) as a positive control. The compounds were diluted at a final concentration of 1 μM using the culture medium IMDM supplemented with 2% (vol/vol) fetal calf serum. This concentration was used to select the most active compounds at the lowest concentration. The wells were filled with 200 μl of a cell suspension containing 20,000 Vero cells and incubated at 37°C for 4 h to adhere. Each well, except eight negative-control wells, then was filled with 50 μl of a parasite suspension containing 60,000 tachyzoites. The plates were incubated at 37°C for 3 h. The wells were emptied to remove any parasite that did not invade host cells. One-hundred-microliter aliquots of diluted compounds were added in the wells, and plates were incubated at 37°C and 5% CO_2_. After 72 h, the cultures were fixed with cold methanol. T. gondii growth was determined on the fixed infected cultures by an enzyme-linked immunosorbent assay (ELISA) using an anti-T. gondii SAG-1-horseradish peroxidase-conjugated monoclonal antibody (Argene Biosoft, France), as previously described (6). Spectrophotometric readings (FLUOstar Omega microplate reader; BMG Labtech, France) were made at 450 nm and corrected at 630 nm, and blank readings were made on the mean value of the seven negative-control wells. For a visual control, the last well of each concentration was stained with kit RAL 555 (RAL Diagnostics, France) and examined microscopically (AxioVert 200M; Zeiss, France) at a magnification of ×20 instead of being used for ELISA. Optical density (OD) values for cultures without drug treatment were used as the 100% value of parasite growth and plotted as a function of the logarithm of each compound concentration.

### Cytotoxicity evaluation.

The *in vitro* cytotoxicity of compounds was evaluated on Vero cell cultures by using 96-well plates, since these cells were used for T. gondii growth in our model. Briefly, 200-μl aliquots of a cell suspension containing 20,000 Vero cells were placed into each well and incubated at 37°C under 5% CO_2_ for 4 h to adhere. Each well, except the eight negative-control wells, then were emptied. They were refilled with 100 μl of each selected effective compound at eight concentrations, obtained by 2-fold dilution series in the culture medium (from 100 to 0.8 μM), except for the eight positive-control wells. Each concentration was assessed in two replicate wells in two replicate plates. After 72 h, cytotoxicity was evaluated by using the UptiBlue viable cell counting assay (Interchim). Wells were emptied and washed with cold phosphate-buffered saline (Sigma-Aldrich, France), and volumes of 100 μl of IMDM supplemented with 2% (vol/vol) fetal calf serum and 10% (vol/vol) UptiBlue were added in each well. Afterwards, the plates were incubated at 37°C and 5% CO_2_ for 3 h. Spectrophotometric measurements (FLUOstar Omega microplate reader; BMG Labtech, France) were made at 570 nm and corrected at 600 nm, and blank readings were made on the mean value of the seven negative-control wells. A sample was considered toxic when the cell viability was lower than 80%. The growth inhibition percentage was calculated from the optical densities relative to the negative control, and 50% cytotoxic concentration (CC_50_) values were determined using Microsoft Excel. For a visual control, the last well of each condition was fixed with cold methanol and stained with kit RAL 555 (RAL Diagnostics, France) and examined microscopically (AxioVert 200M; Zeiss, France) at a magnification of ×20 instead of being tested with UptiBlue.

### Determination of IC_50_s.

The *in vitro* chemosensitivity of T. gondii was assessed by using 96-well plates, as previously described (6), for each compound inhibiting at least 50% of parasite growth at 1 μM. Briefly, 200-μl aliquots of cell suspension containing 20,000 Vero cells were placed into each well and incubated at 37°C and 5% CO_2_ for 4 h to adhere. Each well, except the eight negative-control wells, was filled with 50 μl of a parasite suspension containing 60,000 tachyzoites. The plates were incubated at 37°C and 5% CO_2_ for 3 h. The wells then were emptied to remove any parasite that did not invade host cells. They were refilled with 100 μl of each selected compound at eight concentrations, obtained by 2-fold dilution series in the culture medium (from 2 to 0.015 μM), except for eight positive-control wells. Each concentration was assessed in two replicate wells in two replicate plates. Pyrimethamine was used as a positive control. After 72 h at 37°C and 5% CO_2_, the plates were fixed with cold methanol. The results were obtained by using the same protocol as that previously described for the screening. Each condition was microscopically controlled (AxioVert 200M; Zeiss, France) before the ELISA. The IC_50_s were determined as the sample concentration for which 50% of parasite growth was inhibited. IC_50_s depend on the experimental model (30) and the techniques (9).

### SI.

A selectivity index (SI) was calculated for each compound as the ratio between cytotoxic and antiparasitic activities according to the following formula: SI_T. gondii_ = CC_50Vero_/IC_50T. gondii_.

### Statistical analysis.

For the IC_50_ comparison, a one-way analysis of variance (ANOVA) test followed by a Bonferroni's multiple-comparison test were performed (*P* < 0.05). The software used was GraphPad Prism 6.0.

### Structural similarity measurements.

Molecular fingerprints were used as descriptors in order to structurally compare the active molecules. For this purpose, the chemical structures of the active molecules were encoded into a series of binary digits that represent the presence (1) or absence (0) of substructures within a given molecule. Among the various existing fingerprinting methods (35), atom pair fingerprints (APfp) (36) is among the most popular and has been reported to be the best method to compare close structural analogues (37). Therefore, the APfp was selected to map the molecular structures of PYR and of the eight active molecules presenting the best activity into vectors containing 1,024 bits, where each bit coded for the presence or absence of a particular molecular fragment. The obtained fingerprints were submitted to hierarchical clustering analysis in order to classify the nine molecules. The Tanimoto coefficient, Tc, and Ward's agglomeration method were used for similarity measurements.

Tc is a common fingerprint-based similarity measurement calculation method (38) with the following formula: *S*_A,B_ = *a*/(*a* + *b* − *c*), where *S* represents the similarity between two molecules, A and B, *a* the number of 1 bits in molecule A, *b* the number of 1 bits in molecule B, and *c* the number of common bits. All calculations were performed using the Chemminer package (39) under R.3.3.3. Clustering analysis was performed using the R base stats package, and the gplots package was used for the plots.
